# Supplementary material for: Identification of Freezing-Responsive microRNAs and Their Targets in Chinese Jujube by Small RNA and Degradome Sequencing
Source: Int J Mol Sci. 2026 Sep 4;27(17):7912. doi: 10.3390/ijms27177912 (PMC13566890; doi:10.3390/ijms27177912)
Supplement: Supplementary file 1 [file ijms-27-07912-s001.zip › Supplementary Material S2/novel_miR_132.pdf]

Provisional ID	:	novel_miR_132
Score total	:	0.8
Score for star read(s)	:	-1.3
Score for read counts	:	0
Score for mfe	:	0.5
Score for randfold	:	1.6
Score for cons. seed	:	
Total read count	:	5009
Mature read count	:	2845
Loop read count	:	27
Star read count	:	2137

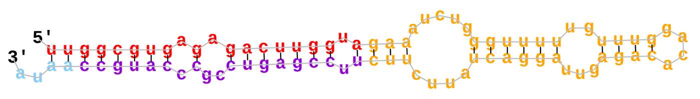

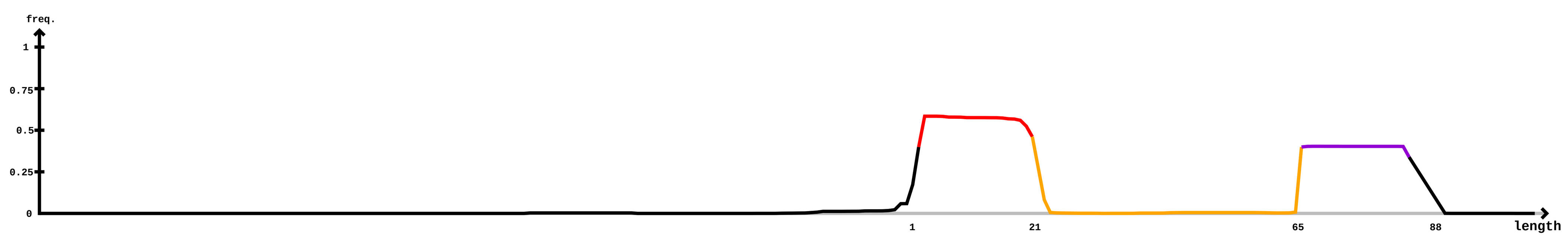

Mature Star

```
5'- ucacgugauaacgcguccuuugcuuuuuauuuuuuauuugaaauucaaaaaaauaauaauaaagauauauuuauauaauuguggaugugaaagaagaacuacuaccacuacacagagugauuuuuuuaugaggcugagaaaauuguuggcgugagagacuugguagaaaucuggguuuuuguuuggacacagaguuaggacuauucuucuuccgaguccgcccaugccaauaauuuccuccgucuuuuuc -3' obs
    ucacgugauaacgcguccuuugcuuuuuauuuuuuauuugaaauucaaaaaaauaauaauaaagauauauuuauauaauuguggaugugaaagaagaacuacuaccacuacacagagugauuuuuuuaugaggcugagaaaauuguuggcgugagagacuugguagaaaucuggguuuuuguuuggacacagaguuaggacuauucuucuuccgaguccgcccaugccaauaauuuccuccgucuuuuuc exp
    ..(((((....)))))......((((((((.(((.(((((...))))).))).))))).)))).((((((((((....))))))))))((((((..(((.((.........)))))..))))))...(((((.(((.((((((((((((((.(.(((((((.((((.....((((((..((((....))))...))))))....)))).)))))))..).)))))))))))))).))).)))))....
```

read	reads	mm	sample
guggaugugaaagaagaa	6	0	JT-2
guggaugugaaagaagaa	9	0	JT-3
gauuuuuuuaugaggcugagaa	1	0	LT-1
uuuuuaugaggcugagaaaau	1	0	JCK-2
uuuuuaugaggcugagaaaau	1	0	JCK-1
uuuuuaugaggcugagaaaauug	3	0	JCK-2
uuuuaugaggcugagaaaauuguuggcgug	2	0	LT-1
uuuaugaggcugagaaaauuguuggcgug	1	0	JT-1
uuaugaggcugagaaaauug	1	0	LT-1
uuaugaggcugagaaaauuguuggcgu	1	0	LT-2
uuaugaggcugagaaaauuguuggcgu	1	0	LT-1
uaugaggcugagaaaauuguuggcgug	2	0	LT-1
augaggcugagaaaauuguuggc	1	0	LT-1
augaggcugagaaaauuguuggU	1	1	LT-2
augaggcugagaaaauuguuggc	2	0	LT-3
augaggcugagaaaauuguuggcg	5	0	JT-2
augaggcugagaaaauuguuggcg	3	0	JT-3
ugaggcugagaaaauuguugg	3	0	LCK-3
ugaggcugagaaaauuAuugg	1	1	JCK-3
ugaggcugagaaaauuguugg	6	0	LT-2
ugaggcugagaaaauuguuggc	1	0	JCK-3
ugaggcugagaaaauuguuggc	1	0	JCK-2
ugaggcugagaaaauuguuggcgug	1	0	JCK-1
ugaggcugagaaaauuguuggcgugaga	1	0	LT-1
gaggcugagaaaauuguuggc	17	0	LCK-3
gaggcugagaaaauuguuggc	1	0	LT-2
gaggcugagaaaauuguuggcg	2	0	LT-3
gaggcugagaaaauuguuggcgug	1	0	JCK-1
gaggcugagaaaauuguuggcgug	2	0	LT-3
gaggcugagaaaauuAuuggcgug	1	1	JCK-2
gaggcugagaaaauuAuuggcgug	2	1	JCK-3
cugagaaaauuguuggcgug	1	0	LT-3
cugagaaaauuguuggcguga	1	0	LT-3

Mature / **Star**

ucacgugauaacgcguccuuugcuuuuuauuuuuuauuugaaauucaaaaaaauaauaauaaagauauauuuauauaauuguggaugugaaagaagaacuacuaccacuacacagagugauuuuuuuaugaggcugagaaaauuguuggcgugagagacuugguagaaaucuggguuuuuguuuggacacagaguuaggacuauucuucuuccgaguccgcccaugccaauaauuucccuccgucuuuuc

ugagaaaauuguuggcgugagagacu	1	0	LT-3
gagaaaauugAuggcgugagaga	1	1	LT-3
agaaaauuguuggcgugagag	3	0	LT-3
agaaaauuguuggcgugagag	2	0	LT-1
agaaaauuguuggcgugagagacu	6	0	LCK-3
gaaaauuAuuggcgugagagacuuggua	1	1	JCK-1
aaauuAuuggcgugagagacuugg	1	1	JCK-1
aauuguuggcgugagagac	6	0	LCK-3
aauuguuggcgugagagac	1	0	LT-1
aCuuguuggcgugagagacu	1	1	LT-3
aauuguuggcgugagagacuugg	1	0	JCK-2
aauuguuggcgugagagacuugg	2	0	JCK-3
auuguuggcgugagagac	2	0	LT-2
auuAuuggcgugagagacu	1	1	JCK-2
auuguuggcgugagagacuug	2	0	LT-1
auuguuggcgugagagacuug	1	0	LT-3
auuguuggcgugagagacuuggua	4	0	LCK-3
auuAuuggcgugagagacuuggua	2	1	JCK-2
auuguuggcgugagagacuuggua	3	0	LT-1
auuguuggcgugagagacuuggua	4	0	LT-3
auuguuggcgugagagacuuggua	1	0	JCK-1
auuguuggcgugagagacuuggua	4	0	LT-2
auuAuuggcgugagagacuuggua	1	1	JCK-1
auuAuuggcgugagagacuuggua	2	1	JCK-3
uuguuggcAugagagacu	1	1	LCK-3
uuguuggcgugagagacu	2	0	LT-1
uuguuggcgugagagacu	3	0	LT-3
uuAuuggcgugagagacu	3	1	JCK-2
uuguuggcgugagagacu	5	0	LCK-3
uuguuggcgugagagacu	2	0	JCK-2
uuAuuggcgugagagacu	1	1	JCK-3
uuAuuggcgugagagacuu	3	1	JCK-3
uuguuggcgugagagacuu	1	0	JCK-1
uuAuuggcgugagagacuu	1	1	JCK-2
uuguuggcgugagagacuu	2	0	LT-1
uuguuggcgugagagacuug	2	0	LT-2
uuAuuggcgugagagacuug	1	1	JCK-2
uuguuggcgugagagacuug	8	0	LCK-3
uuAuuggcgugagagacuugg	41	1	JCK-2
uuAuuggcgugagagacuugg	11	1	JT-1
uuAuuggcgugagagacuugg	29	1	JCK-3
uuguuggcgugagagacuugg	1	0	JCK-3
uuguuggcgugagagacuugg	5	0	LT-1
uuguuggcgugagagacuugg	10	0	LT-2
uuguuggcgugagagacuugg	6	0	LT-3
uuguuggcgugagagacuugg	7	0	LCK-3
uuAuuggcgugagagacuugg	32	1	JCK-1
uuCuuggcgugagagacuugg	1	1	JCK-2
uuguuggcgugagagacuuggu	1	0	JCK-2
uuAuuggcgugagagacuuggu	1	1	JCK-1
uuguuggcgugagagacuuggu	1	0	LT-2
uuguuggcgugagagacuuggu	1	0	JCK-3
uuguuggcgugagagacuuggu	1	0	JCK-1
uuguuggcgugagagacuuggu	1	0	LT-1
uuAuuggcgugagagacuuggua	1	1	JCK-1
uuguuggcgugagagacuuggua	1	0	LT-1
uuAuuggcgugagagacuuggua	3	1	JCK-2
uuguuggcgugagagacuuggua	2	0	LT-3
uuAuuggcgugagagacuuggua	4	1	JCK-3
uguuggcgugagagacuu	1	0	LT-1
uguuggcgugagagacuugg	1	0	LT-1
uguuggcgugagagacuuggua	2	0	LT-3
Auuggcgugagagacuug	7	1	JCK-3
Auuggcgugagagacuug	2	1	JT-1
Auuggcgugagagacuug	12	1	JCK-2
Auuggcgugagagacuug	6	1	JCK-1
Auuggcgugagagacuugg	5	1	JT-3
Auuggcgugagagacuugg	3	1	JCK-3
Auuggcgugagagacuugg	3	1	JCK-1
Auuggcgugagagacuugg	4	1	JCK-2

	Mature		Star				
ucacgugauaacgcguccuuugcuuuuuauuuuuuauuugaaauucaaaaaaauaauaauaaagauauauuuauauaauuguggaugugaaagaagaacuacuaccacuacacagagugauuuuuuuaugaggcugagaaaauug	uuggcgugagagacuuggua	gaaaucuggguuuuuguuuggacacagaguuaggacuauucuuc	uuccgaguccgcccaugcc	aauaauuuccuccgucuuuuc			

Read	Count		Sample
Auuggcgugagagacuugg	7	1	JT-2
Auuggcgugagagacuuggu	14	1	JCK-1
Auuggcgugagagacuuggu	55	1	JCK-2
Cuuggcgugagagacuuggu	1	1	JCK-2
Auuggcgugagagacuuggu	24	1	JCK-3
Auuggcgugagagacuuggu	3	1	JT-3
Auuggcgugagagacuuggu	3	1	JT-2
Auuggcgugagagacuuggua	9	1	JT-1
Cuuggcgugagagacuuggua	2	1	JCK-3
Auuggcgugagagacuuggua	171	1	JCK-2
guuggcgugagagacuuggua	2	0	LT-1
Cuuggcgugagagacuuggua	2	1	JCK-2
Auuggcgugagagacuuggua	81	1	JCK-1
Auuggcgugagagacuuggua	144	1	JCK-3
Auuggcgugagagacuugguag	20	1	JCK-1
Auuggcgugagagacuugguag	18	1	JCK-3
Auuggcgugagagacuugguag	17	1	JCK-2
guuggcgugagagacuugguagaa	1	0	LT-3
uuggcgugagagacuugg	4	0	JCK-3
uuggcgugagagacuugg	2	0	LT-1
uuggcgugagagacuugg	3	0	LT-3
uuggcgugagagacuugg	4	0	LT-2
uuggcgugagagacuugg	2	0	LCK-3
uuggcgugagagacuugg	6	0	JCK-2
uuggcgugagagacuuggu	3	0	LCK-3
uuggcgugagagacuuggu	11	0	JT-1
uNggcgugagagacuuggu	1	1	JCK-2
uuggcgugagagacuuggu	2	0	JT-2
uuggcgugagagacuuggu	20	0	LCK-1
uuggcgugagagaAuuggu	1	1	JT-2
uuggcgugagagacuuggu	23	0	JCK-3
uuggcgugagagacuuggu	1	0	JT-3
uuggcgugagagacuuggu	24	0	LCK-2
uuggcgugagagacuuggu	17	0	LT-2
uuggcgugagagacuuggu	7	0	LT-3
uuggcgugagagacuuggu	15	0	LT-1
uuggcgugagagacuuggu	18	0	JCK-1
uuggcgugagagacuugUu	1	1	JCK-3
uuggcgugagagacuuggu	41	0	JCK-2
uuggcgugagagacuugguU	10	1	JCK-3
uuggcgugagagacuuggua	154	0	JCK-1
uuggcgugagagacuugCua	1	1	JCK-1
uuggcgugagagacCuggua	1	1	JCK-3
uuggcgugagagacuuggua	13	0	LT-1
uuggcgugagagacuuggua	41	0	LCK-3
uuggcgugagagacuuggua	183	0	JCK-3
uuggcgugagagacuuggua	7	0	JT-2
uuggcgugagagaAuuggua	1	1	JCK-3
uuggcgugagagacuuggua	21	0	LT-2
uuggcgugagagacuugguC	1	1	LT-1
uuggcgugagagacuugguC	2	1	LT-3
uuggcgugagagacuugguU	4	1	LCK-3
uuggcgugagaUacuuggua	1	1	LT-2
uuggcgugCgagacuuggua	1	1	JT-1
uuggcgugagagacuuggua	6	0	JT-3
uuggcgugagagacuugguU	4	1	JCK-2
uuggcgugagagaAuuggua	2	1	JCK-1
uuggcgugagagacuuggua	208	0	JCK-2
uuggcgugagagacuugguU	3	1	JCK-1
uuggcgugagaAacuuggua	1	1	JCK-2
uuggcgugagagacuugguU	2	1	LT-3
uuggcgugagagacuuggua	46	0	LT-3
uuggcgugagCgacuuggua	1	1	JCK-2
uuggcgugagagacuugguC	1	1	JCK-2
uuggcgugagCgacuuggua	2	1	JT-1
uuggcgugagagacuuAgua	1	1	JCK-2
uuggcgugagagacuuggua	32	0	JT-1
uuggcgugagagacuugguaA	2	1	JCK-1
uuggcgugagagacuugguag	62	0	JCK-2
uuggcgugagagacuugguag	27	0	JCK-3

Mature Star

ucacgugauaacgcguccuuugcuuuuuauuuuuuauuugaaauucaaaaaaauaauaauaaagauauauuuauauaauuguggaugugaaagaagaacuacuaccacuacacagagugauuuuuuuaugaggcugagaaaauuguuggcgugagagacuugguagaaaucuggguuuuuguuuggacacagaguuaggacuauucuucuuccgaguccgcccaugccaauaauuuccuccgucuuuuc

uuggcgugagagacuugguag	31	0	JCK-1
uuggcgugagagacuugguag	13	0	LT-2
uuggcgugagagacuugguaA	3	1	JCK-3
uuggcgugagagacuugguag	7	0	LT-3
uuggcgugagagacuugguaA	1	1	JCK-2
uuggcgugagCgacuugguag	1	1	JCK-3
uuggcgugagagacuugguag	40	0	JT-1
uuggcgugagaNacuugguag	1	1	JCK-1
uuggcgugagagacuugguag	2	0	LCK-3
uuggcgugagagacuugguag	8	0	LT-1
uuggcgugagagacuugguagC	1	1	JCK-1
uuggcgugagagacuugguagC	1	1	JT-2
uuggcgugagagacuugguagU	14	1	JCK-3
uuggcgugagagacuugguagU	12	1	JCK-2
uuggcgugagagacuugguagU	3	1	LT-1
uuggcgugagagacuugguagC	5	1	JT-3
uuggcgugagagacuugguagU	18	1	JCK-1
uuggcgugagagacuugguagC	1	1	JCK-2
uuggcgugagagacuugguagU	12	1	LT-3
.uggcgugagagacuuggu	6	0	LT-2
.uggcgugagagacuuggA	2	1	JCK-1
.uggcgugagagacuuggu	2	0	LT-1
.uggcgugagagacuuggu	6	0	JCK-2
.uggcgugagagacuuggu	7	0	JCK-3
.uggcgugagagacuuggu	8	0	JCK-1
.uggcgugagagacuugUu	1	1	JCK-2
.uggcgugagagGcuuggu	1	1	LT-3
.uggcgugagagacuuggu	6	0	LT-3
.uggcgugagagacuuggu	10	0	LCK-3
.uggcgugagagacuugguU	6	1	JCK-2
.uggcgugagagacuuggua	41	0	JCK-1
.uggcgugagagacuuggua	47	0	JCK-3
.uggcgugagagacuugguC	4	1	LCK-3
.uggcgugagagacuugguU	7	1	LCK-3
.uggcgugagagacuuggua	12	0	JT-1
.uggcgugagagacuugguU	1	1	LT-3
.uggcgugagagacuugguC	1	1	JCK-2
.uggcgugagagacuugguU	2	1	JCK-1
.uggcgugagagacuuggua	42	0	LCK-3
.uggcgugagagacuuggua	41	0	LT-2
.uggcgugagagacuuggua	30	0	LT-1
.uggcgugagagacuuggua	9	0	JT-2
.uggcgugagagaAuuggua	1	1	LT-2
.uggcgugagagacuugguC	2	1	JCK-1
.uggcgugagagacuuggua	5	0	JT-3
.uggcgugagagacuuggua	51	0	LT-3
.uggcgugagagacuuggua	50	0	JCK-2
.uggcgugagagacuugguag	35	0	JCK-1
.uggcgugagagacuugguag	29	0	JCK-3
.uggcgugagagacuugguag	20	0	LT-2
.uggcgugagagacuugguaA	3	1	LT-2
.uggcAugagagacuugguag	1	1	JCK-1
.uggcgugCgagacuugguag	1	1	JCK-3
.uggcgugagagacuugguag	25	0	JT-1
.uggcgugagagacuugguag	23	0	JT-3
.uggcgugagCgacuugguag	1	1	LT-3
.uggcgugagagacuugguaU	1	1	LT-1
.uggcgugagagacuugguag	38	0	JCK-2
.uggcgugagagaAuugguag	1	1	LT-2
.uggcgugagagacuugguag	8	0	LCK-3
.uggcgugagagacuugguag	13	0	LT-1
.uggcgugagagacuugguag	26	0	LT-3
.uggcgugagagacuugguag	18	0	JT-2
.uggcgugagagacuugguaga	80	0	LT-3
.uggcgugagagacuugguagU	1	1	JCK-2
.uggcgugagagacuugguaga	14	0	JT-3
.uggcgugagagacuugguaga	29	0	JT-1
.uggcgugagagacuugguagU	1	1	LT-3
.uggcgugagagCcuugguaga	1	1	JCK-3
.uggcgugagagacuugguaga	6	0	JT-2

Mature Star

ucacgugauaacgcguccuuugcuuuuuauuuuuuauuugaaauucaaaaaaauaauaauaaagauauauuuauauaauuguggaugugaaagaagaacuacuaccacuacacagagugauuuuuuuaugaggcugagaaaauuguuggcgugagagacuugguagaaaucuggguuuuuguuuggacacagaguuaggacuauucuucuuccgaguccgcccaugccaauaauuuccuccgucuuuuc

uggcgugagagacuugguaga	54	0	LT-2
uggcgugagagacuugguaga	22	0	JCK-1
uggcgugagagacuugguaga	42	0	LT-1
uggcgugagagacuugguaga	36	0	LCK-3
uggcgugagagacuugguagC	1	1	JCK-3
uggcgugagagacuugguaga	25	0	JCK-3
uggcgugagagacuugguagC	1	1	LT-3
uggcgugCgagacuugguaga	1	1	LT-1
uggcgugagagacuugguaga	27	0	JCK-2
uggcgugagagacuugguagaU	4	1	LT-3
uggcgugagagacuugguagaU	4	1	LT-2
uggcgugagagacuugguagaa	2	0	JCK-1
uggcgugagagacuugguagaa	1	0	JCK-2
uggcgugagagacuugguagaa	1	0	LT-1
cgugagagacuugguaga	1	0	JCK-3
cgugagagacuugguagaaau	1	0	LT-2
cgugagagacuugguagaaauc	1	0	LT-3
cgugagagacuugguagaaaucu	1	0	LT-1
gugagagacuugguagaaauc	2	0	LT-2
ugagagacuugguagaaa	3	0	LT-3
ugagagacuugguagaaa	1	0	LT-1
ugagagacuugguagaaau	1	0	LT-3
ugagagacuugguagaaaucu	1	0	JCK-2
ugagagacuugguagaaaucuggg	2	0	JCK-2
ugagagacuugguagaaaucugggu	1	0	LT-1
acuugguagaaaucugggu	3	0	LT-3
uuuguuuggacacagaguuaggacuau	1	0	JCK-3
uuuguuuggacacagaguuaggacuau	1	0	JCK-2
uuuggacacagaUuuaggacuauu	1	1	LT-3
uuggacacagaUuuaggacu	1	1	LT-1
uuggacacagaguuaggacu	1	0	JCK-3
uuggacacagaUuuaggacua	1	1	LT-1
uuggacacagaUuuaggacua	2	1	LT-2
uuggacacagaguuaggacuau	3	0	LCK-3
acacagaUuuaggacuauucuu	1	1	LT-3
cacagaUuuaggacuauu	2	1	LT-3
cacagaUuuaggacuauucu	2	1	LT-1
cacagaUuuaggacuauucuu	2	1	JCK-3
cacagaUuuaggacuauucuu	1	1	LT-3
cacagaUuuaggacuauucuu	1	1	JCK-1
cacagaUuuaggacuauucuuc	1	1	JCK-3
cacagaguuaggacuauucuucuucc	1	0	LT-3
acagaUuuaggacuauucuucu	2	1	JCK-1
acagaUuuaggacuauucuucu	1	1	JCK-3
cagaUuuaggacuauucuucu	1	1	JCK-3
cagaguuaggacuauucuucuuccgag	1	0	LT-1
uucuuccgaguccgcccaugcc	1	0	LT-3
ucuuccgaguccgcccaug	1	0	JCK-2
ucuuccgaguccgcccaug	3	0	LCK-3
cuuccgaguccgcccaugc	1	0	LT-3
cuuccgaguccgcccaugU	2	1	LT-1
cuuccgaguccgcccaugc	3	0	LCK-3
cuuccgaguccgcccaugc	1	0	LT-1
cuuccgaguccgcccaugc	2	0	LT-2
cuuccgaguccgcccaugcc	3	0	LT-2
cuuccgaguccgcccaugcc	2	0	LT-1
cuuccgaguccgcccaugcc	5	0	LCK-3
cuuccgaguccgcccaugcc	1	0	LT-3
cuuccgaguccgcccaugcca	1	0	LCK-3
cuuccgaguccgcccaugccaaua	3	0	JT-3
cuuccgaguccgcccaugccaaua	5	0	JT-2
uuccgaguccgcccaugc	2	0	JT-2
uuccgaguccgcccaugU	1	1	LT-1
uuccgaguccgcccaugc	12	0	JT-1
uuccgaguccgcccaugc	51	0	LT-1
uuccgaguccgcccaugc	8	0	JCK-1
uuccgaguccgcccaugc	5	0	JT-3
uuccgaguccgcccaugc	97	0	LCK-3
uuccgaguccgcccaugc	5	0	LCK-2
uuccgaguccgcccaugc	88	0	LT-3

Mature Star

```
ucacgugauaacgcguccuuugcuuuuuauuuuuuauuugaaauucaaaaaaauaauaauaaagauauauuuauauaauuguggaugugaaagaagaacuacuaccacuacacagagugauuuuuuuuaugaggcugagaaaauuguuggcgugagagacuugguagaaaucuggguuuuuguuuggacacagaguuaggacuauucuucuuccgaguccgcccaugccaauaauuuccuccgucuuuuc
..................................................................................................................................................................................................................uuccgaguccgcccaugc......................   11   0   LCK-1
..................................................................................................................................................................................................................uuccgaguccgcccaugc......................   10   0   JCK-2
..................................................................................................................................................................................................................uuccgaguccgcccaugc......................   44   0   LT-2
..................................................................................................................................................................................................................uuccgaguccgcccaugc......................   3    0   JCK-3
..................................................................................................................................................................................................................uuccgaguccgcccaugcc.....................   41   0   JCK-2
..................................................................................................................................................................................................................uuccgaguccgcccaugcc.....................   36   0   JCK-3
..................................................................................................................................................................................................................uNccgaguccgcccaugcc.....................   1    1   JCK-1
..................................................................................................................................................................................................................uuccgaguccgcccaugcc.....................   14   0   JT-3
..................................................................................................................................................................................................................uuccgaguccgcccaugcA.....................   1    1   LT-2
..................................................................................................................................................................................................................uuccgaguccgcccaugcc.....................   119  0   LT-1
..................................................................................................................................................................................................................uuccgaguccgcccaugcc.....................   186  0   LT-3
..................................................................................................................................................................................................................uuccgaguccgcccaugcc.....................   10   0   JT-2
..................................................................................................................................................................................................................uuccgaguccgcccaugcU.....................   1    1   JCK-2
..................................................................................................................................................................................................................uuccgaguccgcccaugcc.....................   160  0   LCK-3
..................................................................................................................................................................................................................uuccgaguccgcccaugcU.....................   6    1   LT-3
..................................................................................................................................................................................................................uuccgaguccgcccaugcU.....................   10   1   LCK-3
..................................................................................................................................................................................................................uuccgaguccgcccaugcU.....................   16   1   LT-2
..................................................................................................................................................................................................................uuccgaguccgcccaugcc.....................   9    0   JT-1
..................................................................................................................................................................................................................uuccgCguccgcccaugcc.....................   1    1   LCK-3
..................................................................................................................................................................................................................uuccgaguccgcccaugcc.....................   29   0   JCK-1
..................................................................................................................................................................................................................uuccgaguccgcccaugcU.....................   2    1   JCK-1
..................................................................................................................................................................................................................uuccgaguccgcccaugcU.....................   8    1   LT-1
..................................................................................................................................................................................................................uuccgaguccgcccaugcc.....................   108  0   LT-2
..................................................................................................................................................................................................................uuccgaguccgcccaugccU....................   1    1   LT-3
..................................................................................................................................................................................................................uuccgaguccgcccaugcca....................   28   0   JCK-2
..................................................................................................................................................................................................................uuccgCguccgcccaugcca....................   1    1   JT-1
..................................................................................................................................................................................................................uuccgaguccgcccaugcca....................   59   0   LCK-3
..................................................................................................................................................................................................................uuccgaguccgcccaugccU....................   1    1   LT-2
..................................................................................................................................................................................................................uuccgaguccgcccaugcca....................   7    0   JT-3
..................................................................................................................................................................................................................uuccgaguccgcccaugcca....................   33   0   LT-2
..................................................................................................................................................................................................................uuccgaguccgcccaugccC....................   1    1   LT-2
..................................................................................................................................................................................................................uuccgaguccgcccaugcUa....................   1    1   LT-1
..................................................................................................................................................................................................................uuccgaguccgcccaugcca....................   40   0   LT-1
..................................................................................................................................................................................................................uuccgaguccgcccaugcca....................   19   0   JCK-3
..................................................................................................................................................................................................................uuccgaguccgcccaugcca....................   16   0   JCK-1
..................................................................................................................................................................................................................uuccgaguccgcccaugccC....................   1    1   JCK-2
..................................................................................................................................................................................................................uuccgaguccgcccaugcca....................   37   0   JT-1
..................................................................................................................................................................................................................uuccgaguccgcccaugcca....................   65   0   LT-3
..................................................................................................................................................................................................................uuccgaguccgcccCugcca....................   1    1   LT-2
..................................................................................................................................................................................................................uuccgaguccgcccaugcca....................   1    0   JT-2
..................................................................................................................................................................................................................uuccgaguccgcccaugccaa...................   13   0   LT-2
..................................................................................................................................................................................................................uuccgaguccgcccaugccaa...................   24   0   LCK-3
..................................................................................................................................................................................................................uuccgaguccgcccaugccaU...................   1    1   LT-1
..................................................................................................................................................................................................................uuccgaguccgcccaugccaa...................   3    0   JCK-2
..................................................................................................................................................................................................................uuccgaguccgcccaugccaa...................   19   0   LCK-2
..................................................................................................................................................................................................................uuccgaguccgcccaugccaa...................   1    0   JCK-3
..................................................................................................................................................................................................................uuccgaguccgcccaugccaa...................   11   0   LT-1
..................................................................................................................................................................................................................uuccgaguccgcccaugccaa...................   12   0   LT-3
..................................................................................................................................................................................................................uuccgaguccgcccaugccaa...................   16   0   LCK-1
..................................................................................................................................................................................................................uuccgaguccgcccaugccaa...................   2    0   JCK-1
..................................................................................................................................................................................................................uuccgaguccgcccaugccaau..................   26   0   LCK-1
..................................................................................................................................................................................................................uuccgCguccgcccaugccaau..................   2    1   JCK-2
..................................................................................................................................................................................................................uuccgaguccgcccaugcAaau..................   1    1   JCK-3
..................................................................................................................................................................................................................uuccgaguccgcccaugccaau..................   19   0   LT-2
..................................................................................................................................................................................................................uuccgaguccgcccaugccaau..................   48   0   LT-1
..................................................................................................................................................................................................................uuccgCguccgcccaugccaau..................   1    1   JCK-3
..................................................................................................................................................................................................................uuccgaguccgcccaugccaau..................   62   0   LT-3
..................................................................................................................................................................................................................uuccgaguccgcccaugccaau..................   14   0   LCK-2
..................................................................................................................................................................................................................uuccgaguccgcccaugccaau..................   76   0   JCK-1
..................................................................................................................................................................................................................uuccgaguccgcccaugccUau..................   1    1   JCK-1
..................................................................................................................................................................................................................uuccgaguccgcccaugccaau..................   10   0   LCK-3
..................................................................................................................................................................................................................uucUgaguccgcccaugccaau..................   1    1   JCK-3
..................................................................................................................................................................................................................uuccgaguccgcccaugccaau..................   91   0   JCK-3
..................................................................................................................................................................................................................uuccgaguccgcccaugccaau..................   115  0   JCK-2
..................................................................................................................................................................................................................uuccgaguccgcccaugccaCu..................   1    1   JCK-2
..................................................................................................................................................................................................................uuccgaguccgcccaugccUau..................   2    1   JCK-2
..................................................................................................................................................................................................................uuccgaguccgcccaugccaauU.................   27   1   JCK-3
..................................................................................................................................................................................................................uuccgaguccgcccaugccaauU.................   4    1   LCK-3
..................................................................................................................................................................................................................uuccgaguccgcccaugccaaua.................   1    0   LT-2
..................................................................................................................................................................................................................uuccgaguccgcccaugccaauU.................   24   1   JCK-2
```

Mature Star

```
ucacgugauaacgcguccuuugcuuuuuauuuuuuauuugaaauucaaaaaaauaauaauaaagauauauuuauauaauuguggaugugaaagaagaacuacuaccacuacacagagugauuuuuuuaugaggcugagaaaauuguuggcgugagagacuugguagaaaucuggguuuuuguuuggacacagaguuaggacuauucuucuuccgaguccgcccaugccaauaauuuccuccgucuuuuc
.................................................................................................................................................................................................................uuccgaguccgcccaugccaauU.................   17   1   LT-3
.................................................................................................................................................................................................................uuccgaguccgcccaugccaauU.................   8    1   LT-2
.................................................................................................................................................................................................................uuccgaguccgcccaugccaauU.................   16   1   JCK-1
.................................................................................................................................................................................................................uuccgaguccgcccaugccaauU.................   23   1   LT-1
.................................................................................................................................................................................................................uuccgaguccgcccaugccaauUa................   2    1   JCK-1
.................................................................................................................................................................................................................uuccgaguccgcccaugccaauUa................   1    1   LT-3
.................................................................................................................................................................................................................uuccgaguccgcccaugccaauUa................   3    1   JCK-3
.................................................................................................................................................................................................................uuccgaguccgcccaugccaauUa................   4    1   JCK-2
.................................................................................................................................................................................................................uuccgaguccgcccaugccaauaUu...............   1    1   LT-1
.................................................................................................................................................................................................................uuccgaguccgcccaugccaauUau...............   1    1   LT-1
..................................................................................................................................................................................................................uccgaguccgcccaugcc.....................   1    0   LT-1
..................................................................................................................................................................................................................uccgaguccgcccaugcc.....................   1    0   JCK-3
..................................................................................................................................................................................................................uccgaguccgcccaugcca....................   1    0   LT-1
..................................................................................................................................................................................................................uccgaguccgcccaugcca....................   1    0   JCK-3
..................................................................................................................................................................................................................uccgaguccgcccaugccaa...................   2    0   LT-2
..................................................................................................................................................................................................................uccgaguccgcccaugccaau..................   2    0   JCK-3
..................................................................................................................................................................................................................uccgaguccgcccaugccaau..................   2    0   JCK-2
..................................................................................................................................................................................................................uccgaguccgcccaugccaau..................   1    0   LT-2
..................................................................................................................................................................................................................uccgaguccgcccaugccaauU.................   1    1   JCK-2
..................................................................................................................................................................................................................uccgaguccgcccaugccaaua.................   2    0   JCK-3
..................................................................................................................................................................................................................uccgaguccgcccaugccaaua.................   5    0   JCK-2
..................................................................................................................................................................................................................uccgaguccgcccaugccaaua.................   4    0   JCK-1
...................................................................................................................................................................................................................ccgaguccgcccaugccaaua.................   1    0   JCK-2
...................................................................................................................................................................................................................ccgaguccgcccaugccaaua.................   2    0   JCK-3
..................................................................................................................................................................................................................................ccaauaauuuccuccgucu.....   1    0   LT-3
```
